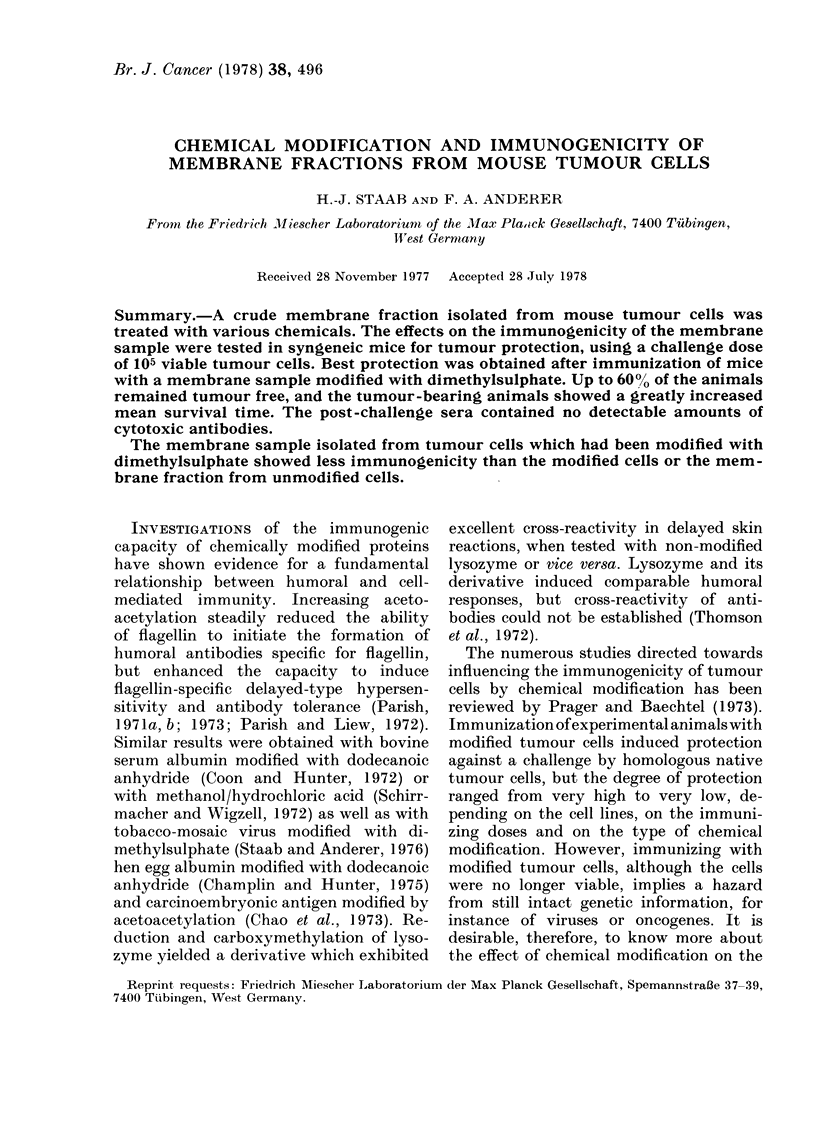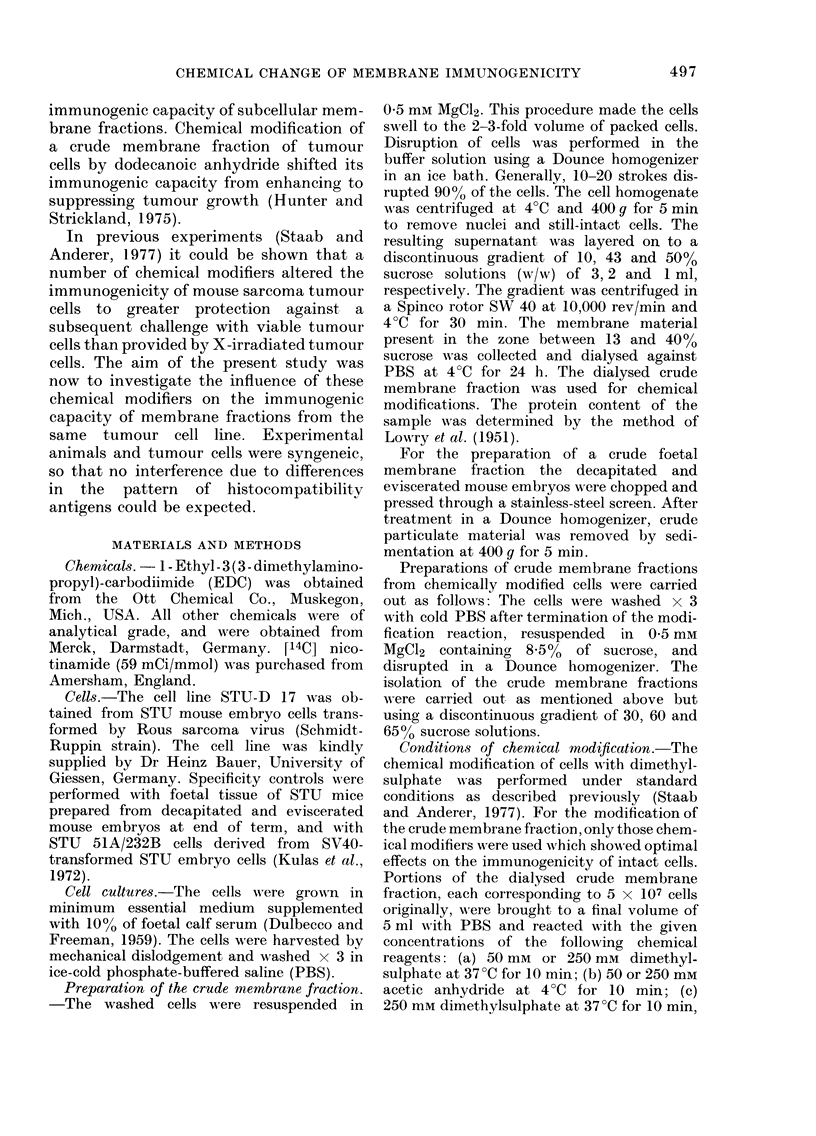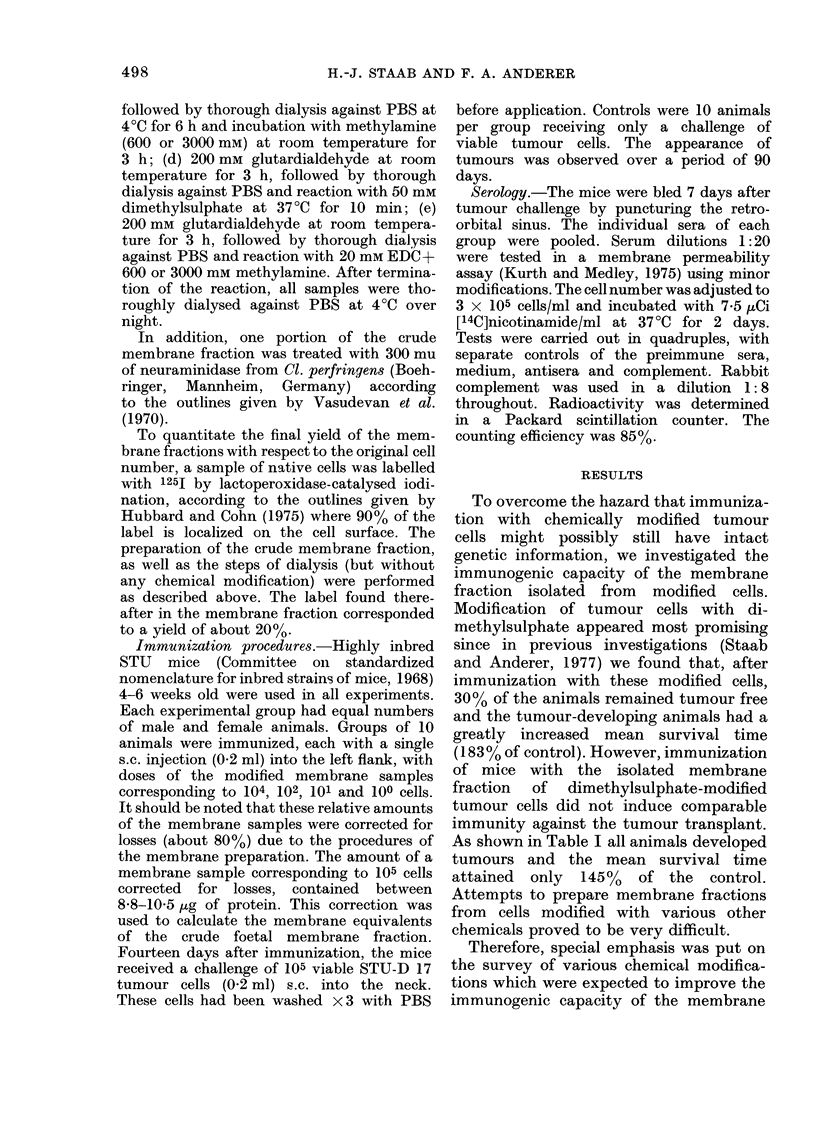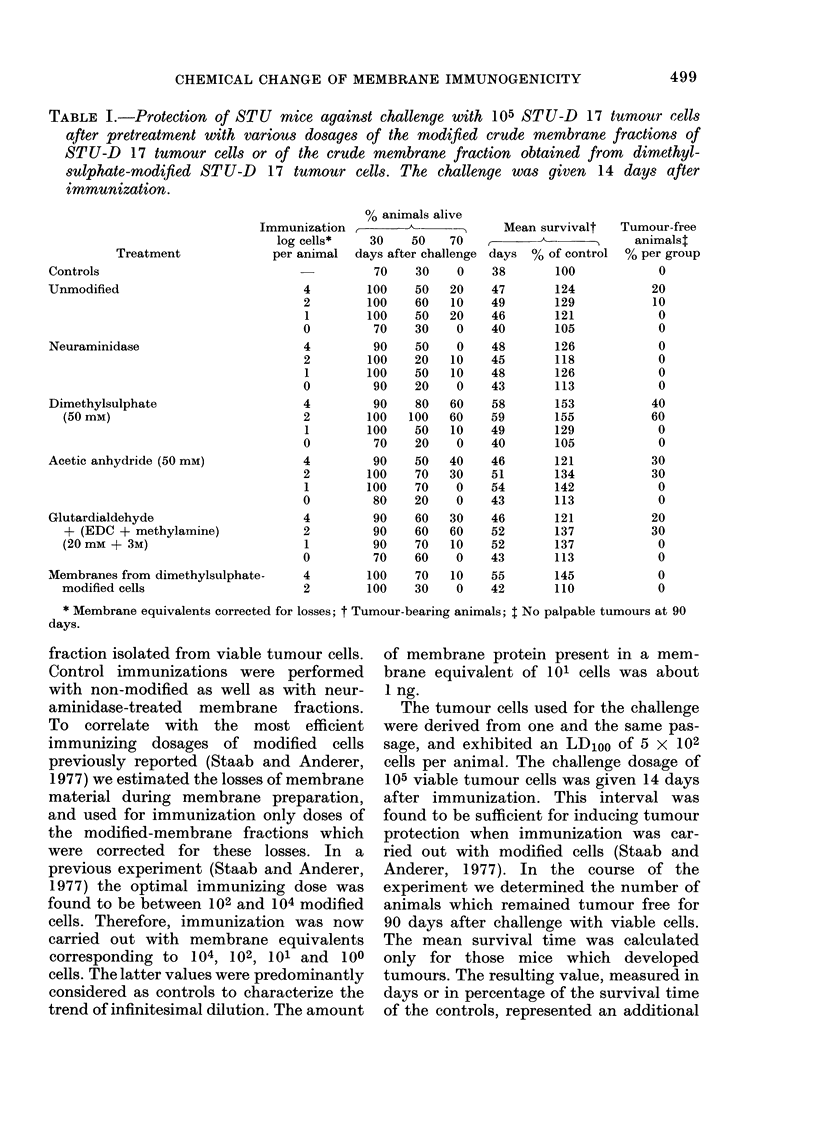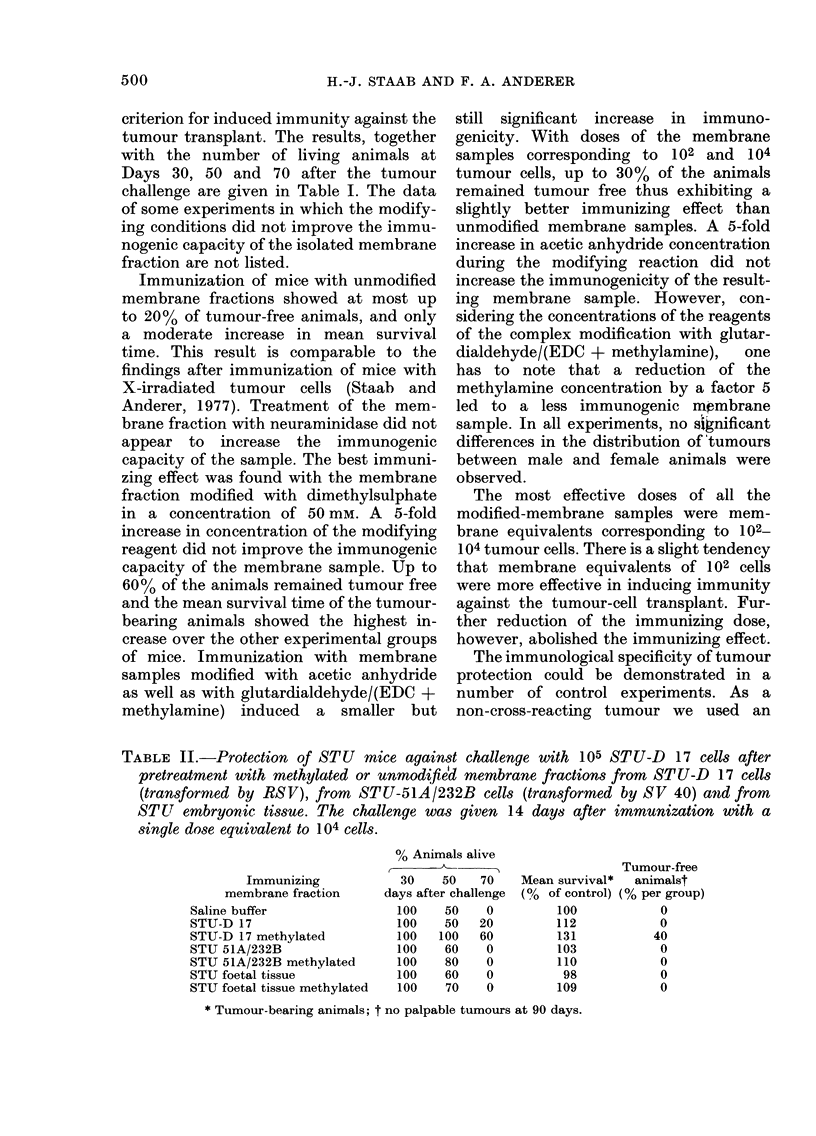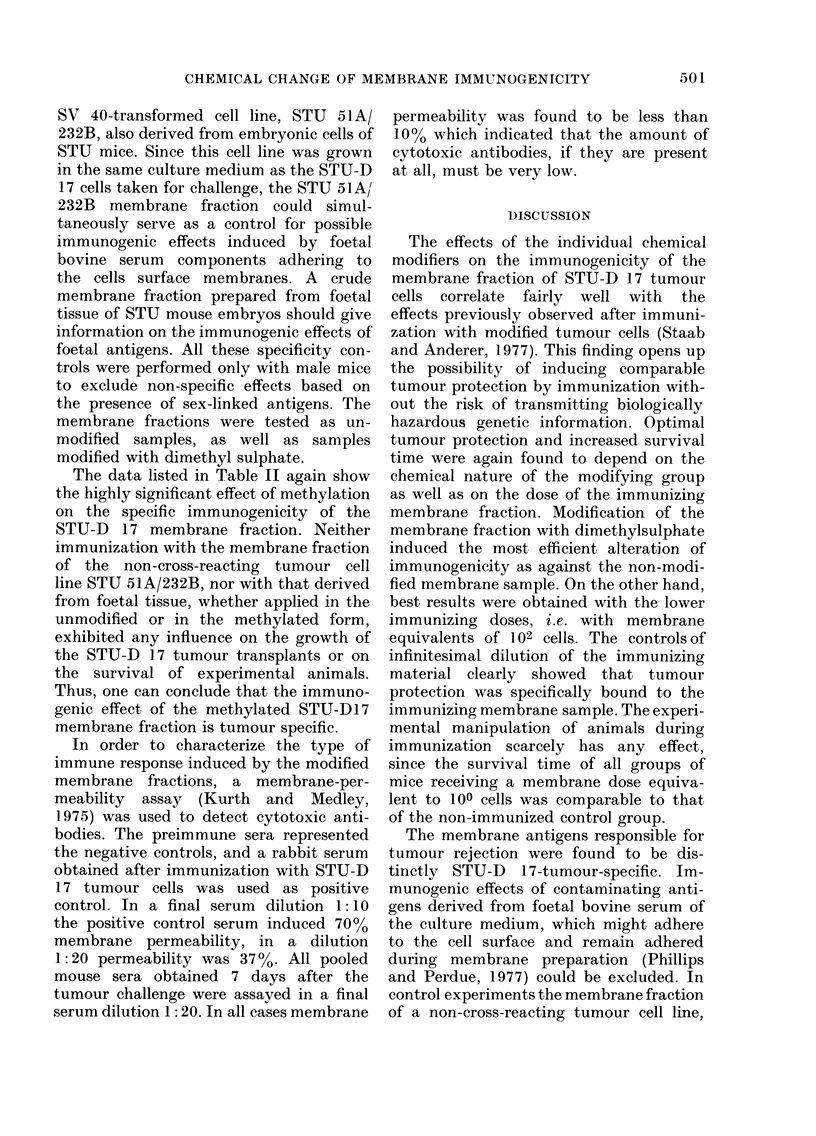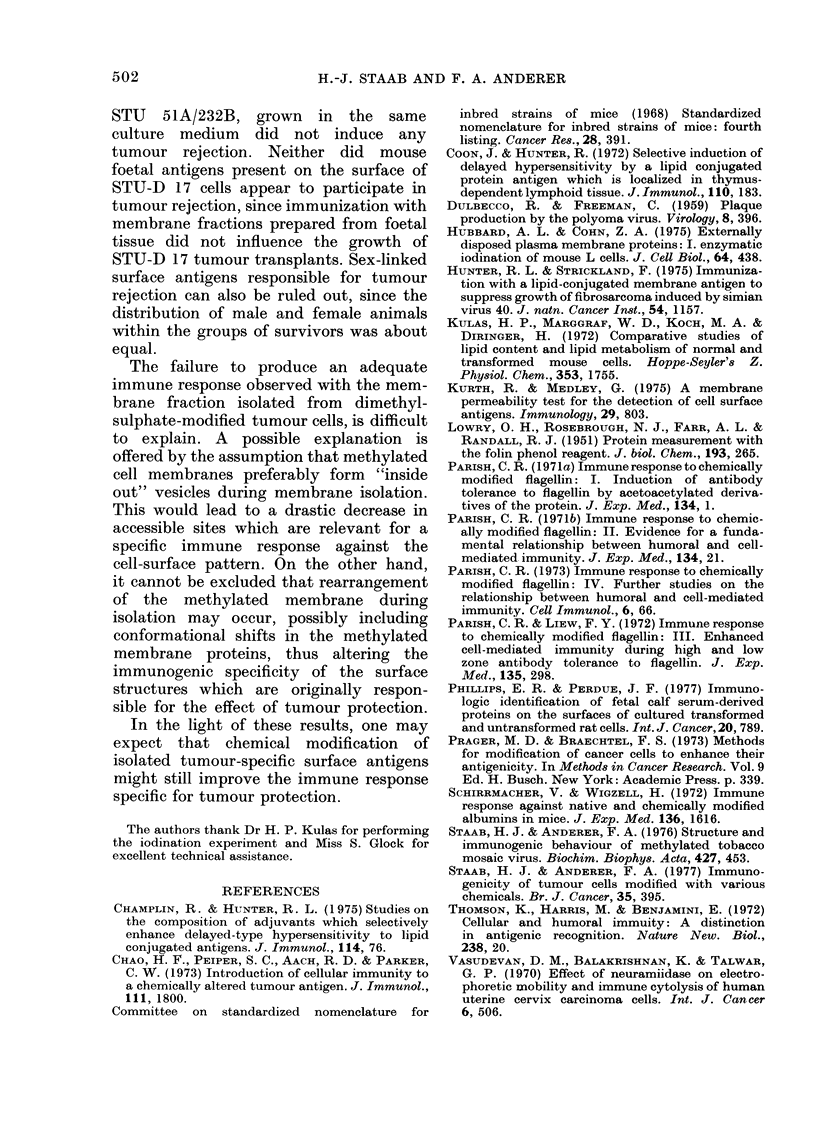# Chemical modification and immunogenicity of membrane fractions from mouse tumour cells.

**DOI:** 10.1038/bjc.1978.236

**Published:** 1978-10

**Authors:** H. J. Staab, F. A. Anderer

## Abstract

A crude membrane fraction isolated from mouse tumour cells was treated with various chemicals. The effects on the immunogenicity of the membrane sample were tested in syngeneic mice for tumour protection, using a challenge dose of 10(5) viable tumour cells. Best protection was obtained after immunization of mice with a membrane sample modified with dimethylsulphate. Up to 60% of the animals remained tumour free, and the tumour-bearing animals showed a greatly increased mean survival time. The post-challenge sera contained no detectable amounts of cytotoxic antibodies. The membrane sample isolated from tumour cells which had been modified with dimethylsulphate showed less immunogenicity than the modified cells or the membrane fraction from unmodified cells.


*Br. J. Cancer* (1978) **38**, 496

### CHEMICAL MODIFICATION AND IMMUNOGENICITY OF MEMBRANE FRACTIONS FROM MOUSE TUMOUR CELLS

H.-J. STAAB AND F. A. ANDERER

*From the Friedrich Miescher Laboratorium of the Max Planck Gesellschaft,* 7400 *Tübingen, West Germany*

Received 28 November 1977 Accepted 28 July 1978

**Summary.—A crude membrane fraction isolated from mouse tumour cells was treated with various chemicals. The effects on the immunogenicity of the membrane sample were tested in syngeneic mice for tumour protection, using a challenge dose of $10^5$ viable tumour cells. Best protection was obtained after immunization of mice with a membrane sample modified with dimethylsulphate. Up to 60% of the animals remained tumour free, and the tumour-bearing animals showed a greatly increased mean survival time. The post-challenge sera contained no detectable amounts of cytotoxic antibodies.**

**The membrane sample isolated from tumour cells which had been modified with dimethylsulphate showed less immunogenicity than the modified cells or the membrane fraction from unmodified cells.**

INVESTIGATIONS of the immunogenic capacity of chemically modified proteins have shown evidence for a fundamental relationship between humoral and cell-mediated immunity. Increasing aceto-acetylation steadily reduced the ability of flagellin to initiate the formation of humoral antibodies specific for flagellin, but enhanced the capacity to induce flagellin-specific delayed-type hypersensitivity and antibody tolerance (Parish, 1971*a*, *b*; 1973; Parish and Liew, 1972). Similar results were obtained with bovine serum albumin modified with dodecanoic anhydride (Coon and Hunter, 1972) or with methanol/hydrochloric acid (Schirrmacher and Wigzell, 1972) as well as with tobacco-mosaic virus modified with dimethylsulphate (Staab and Anderer, 1976) hen egg albumin modified with dodecanoic anhydride (Champlin and Hunter, 1975) and carcinoembryonic antigen modified by acetoacetylation (Chao *et al.*, 1973). Reduction and carboxymethylation of lysozyme yielded a derivative which exhibited excellent cross-reactivity in delayed skin reactions, when tested with non-modified lysozyme or *vice versa*. Lysozyme and its derivative induced comparable humoral responses, but cross-reactivity of antibodies could not be established (Thomson *et al.*, 1972).

The numerous studies directed towards influencing the immunogenicity of tumour cells by chemical modification has been reviewed by Prager and Baechtel (1973). Immunization of experimental animals with modified tumour cells induced protection against a challenge by homologous native tumour cells, but the degree of protection ranged from very high to very low, depending on the cell lines, on the immunizing doses and on the type of chemical modification. However, immunizing with modified tumour cells, although the cells were no longer viable, implies a hazard from still intact genetic information, for instance of viruses or oncogenes. It is desirable, therefore, to know more about the effect of chemical modification on the

Reprint requests: Friedrich Miescher Laboratorium der Max Planck Gesellschaft, Spemannstraße 37–39, 7400 Tübingen, West Germany.

immunogenic capacity of subcellular membrane fractions. Chemical modification of a crude membrane fraction of tumour cells by dodecanoic anhydride shifted its immunogenic capacity from enhancing to suppressing tumour growth (Hunter and Strickland, 1975).

In previous experiments (Staab and Anderer, 1977) it could be shown that a number of chemical modifiers altered the immunogenicity of mouse sarcoma tumour cells to greater protection against a subsequent challenge with viable tumour cells than provided by X-irradiated tumour cells. The aim of the present study was now to investigate the influence of these chemical modifiers on the immunogenic capacity of membrane fractions from the same tumour cell line. Experimental animals and tumour cells were syngeneic, so that no interference due to differences in the pattern of histocompatibility antigens could be expected.

### MATERIALS AND METHODS

*Chemicals.*—1-Ethyl-3(3-dimethylamino-propyl)-carbodiimide (EDC) was obtained from the Ott Chemical Co., Muskegon, Mich., USA. All other chemicals were of analytical grade, and were obtained from Merck, Darmstadt, Germany. [$^{14}$C] nicotinamide (59 mCi/mmol) was purchased from Amersham, England.

*Cells.*—The cell line STU-D 17 was obtained from STU mouse embryo cells transformed by Rous sarcoma virus (Schmidt-Ruppin strain). The cell line was kindly supplied by Dr Heinz Bauer, University of Giessen, Germany. Specificity controls were performed with foetal tissue of STU mice prepared from decapitated and eviscerated mouse embryos at end of term, and with STU 51A/232B cells derived from SV40-transformed STU embryo cells (Kulas *et al.*, 1972).

*Cell cultures.*—The cells were grown in minimum essential medium supplemented with 10% of foetal calf serum (Dulbecco and Freeman, 1959). The cells were harvested by mechanical dislodgement and washed × 3 in ice-cold phosphate-buffered saline (PBS).

*Preparation of the crude membrane fraction.*—The washed cells were resuspended in 0·5 mM $MgCl_2$. This procedure made the cells swell to the 2–3-fold volume of packed cells. Disruption of cells was performed in the buffer solution using a Dounce homogenizer in an ice bath. Generally, 10–20 strokes disrupted 90% of the cells. The cell homogenate was centrifuged at 4°C and 400 *g* for 5 min to remove nuclei and still-intact cells. The resulting supernatant was layered on to a discontinuous gradient of 10, 43 and 50% sucrose solutions (w/w) of 3, 2 and 1 ml, respectively. The gradient was centrifuged in a Spinco rotor SW 40 at 10,000 rev/min and 4°C for 30 min. The membrane material present in the zone between 13 and 40% sucrose was collected and dialysed against PBS at 4°C for 24 h. The dialysed crude membrane fraction was used for chemical modifications. The protein content of the sample was determined by the method of Lowry *et al.* (1951).

For the preparation of a crude foetal membrane fraction the decapitated and eviscerated mouse embryos were chopped and pressed through a stainless-steel screen. After treatment in a Dounce homogenizer, crude particulate material was removed by sedimentation at 400 *g* for 5 min.

Preparations of crude membrane fractions from chemically modified cells were carried out as follows: The cells were washed × 3 with cold PBS after termination of the modification reaction, resuspended in 0·5 mM $MgCl_2$ containing 8·5% of sucrose, and disrupted in a Dounce homogenizer. The isolation of the crude membrane fractions were carried out as mentioned above but using a discontinuous gradient of 30, 60 and 65% sucrose solutions.

*Conditions of chemical modification.*—The chemical modification of cells with dimethylsulphate was performed under standard conditions as described previously (Staab and Anderer, 1977). For the modification of the crude membrane fraction, only those chemical modifiers were used which showed optimal effects on the immunogenicity of intact cells. Portions of the dialysed crude membrane fraction, each corresponding to 5 × $10^7$ cells originally, were brought to a final volume of 5 ml with PBS and reacted with the given concentrations of the following chemical reagents: (a) 50 mM or 250 mM dimethylsulphate at 37°C for 10 min; (b) 50 or 250 mM acetic anhydride at 4°C for 10 min; (c) 250 mM dimethylsulphate at 37°C for 10 min, followed by thorough dialysis against PBS at 4 °C for 6 h and incubation with methylamine (600 or 3000 mM) at room temperature for 3 h; (d) 200 mM glutardialdehyde at room temperature for 3 h, followed by thorough dialysis against PBS and reaction with 50 mM dimethylsulphate at 37 °C for 10 min; (e) 200 mM glutardialdehyde at room temperature for 3 h, followed by thorough dialysis against PBS and reaction with 20 mM EDC+600 or 3000 mM methylamine. After termination of the reaction, all samples were thoroughly dialysed against PBS at 4 °C over night.

In addition, one portion of the crude membrane fraction was treated with 300 mu of neuraminidase from *Cl. perfringens* (Boehringer, Mannheim, Germany) according to the outlines given by Vasudevan *et al.* (1970).

To quantitate the final yield of the membrane fractions with respect to the original cell number, a sample of native cells was labelled with $^{125}I$ by lactoperoxidase-catalysed iodination, according to the outlines given by Hubbard and Cohn (1975) where 90% of the label is localized on the cell surface. The preparation of the crude membrane fraction, as well as the steps of dialysis (but without any chemical modification) were performed as described above. The label found thereafter in the membrane fraction corresponded to a yield of about 20%.

*Immunization procedures.*—Highly inbred STU mice (Committee on standardized nomenclature for inbred strains of mice, 1968) 4–6 weeks old were used in all experiments. Each experimental group had equal numbers of male and female animals. Groups of 10 animals were immunized, each with a single s.c. injection (0·2 ml) into the left flank, with doses of the modified membrane samples corresponding to $10^4$, $10^2$, $10^1$ and $10^0$ cells. It should be noted that these relative amounts of the membrane samples were corrected for losses (about 80%) due to the procedures of the membrane preparation. The amount of a membrane sample corresponding to $10^5$ cells corrected for losses, contained between 8·8–10·5 μg of protein. This correction was used to calculate the membrane equivalents of the crude foetal membrane fraction. Fourteen days after immunization, the mice received a challenge of $10^5$ viable STU-D 17 tumour cells (0·2 ml) s.c. into the neck. These cells had been washed ×3 with PBS before application. Controls were 10 animals per group receiving only a challenge of viable tumour cells. The appearance of tumours was observed over a period of 90 days.

*Serology.*—The mice were bled 7 days after tumour challenge by puncturing the retro-orbital sinus. The individual sera of each group were pooled. Serum dilutions 1:20 were tested in a membrane permeability assay (Kurth and Medley, 1975) using minor modifications. The cell number was adjusted to $3 \times 10^5$ cells/ml and incubated with 7·5 μCi [$^{14}C$]nicotinamide/ml at 37 °C for 2 days. Tests were carried out in quadruples, with separate controls of the preimmune sera, medium, antisera and complement. Rabbit complement was used in a dilution 1:8 throughout. Radioactivity was determined in a Packard scintillation counter. The counting efficiency was 85%.

### RESULTS

To overcome the hazard that immunization with chemically modified tumour cells might possibly still have intact genetic information, we investigated the immunogenic capacity of the membrane fraction isolated from modified cells. Modification of tumour cells with dimethylsulphate appeared most promising since in previous investigations (Staab and Anderer, 1977) we found that, after immunization with these modified cells, 30% of the animals remained tumour free and the tumour-developing animals had a greatly increased mean survival time (183% of control). However, immunization of mice with the isolated membrane fraction of dimethylsulphate-modified tumour cells did not induce comparable immunity against the tumour transplant. As shown in Table I all animals developed tumours and the mean survival time attained only 145% of the control. Attempts to prepare membrane fractions from cells modified with various other chemicals proved to be very difficult.

Therefore, special emphasis was put on the survey of various chemical modifications which were expected to improve the immunogenic capacity of the membrane

TABLE I.—*Protection of STU mice against challenge with* $10^5$ *STU-D* 17 *tumour cells after pretreatment with various dosages of the modified crude membrane fractions of STU-D* 17 *tumour cells or of the crude membrane fraction obtained from dimethylsulphate-modified STU-D* 17 *tumour cells. The challenge was given* 14 *days after immunization.*

| Treatment | Immunization log cells* per animal | % animals alive 30 days after challenge | 50 | 70 | Mean survival† days | % of control | Tumour-free animals‡ % per group |
|---|---|---|---|---|---|---|---|
| Controls | — | 70 | 30 | 0 | 38 | 100 | 0 |
| Unmodified | 4 | 100 | 50 | 20 | 47 | 124 | 20 |
| | 2 | 100 | 60 | 10 | 49 | 129 | 10 |
| | 1 | 100 | 50 | 20 | 46 | 121 | 0 |
| | 0 | 70 | 30 | 0 | 40 | 105 | 0 |
| Neuraminidase | 4 | 90 | 50 | 0 | 48 | 126 | 0 |
| | 2 | 100 | 20 | 10 | 45 | 118 | 0 |
| | 1 | 100 | 50 | 10 | 48 | 126 | 0 |
| | 0 | 90 | 20 | 0 | 43 | 113 | 0 |
| Dimethylsulphate | 4 | 90 | 80 | 60 | 58 | 153 | 40 |
| (50 mM) | 2 | 100 | 100 | 60 | 59 | 155 | 60 |
| | 1 | 100 | 50 | 10 | 49 | 129 | 0 |
| | 0 | 70 | 20 | 0 | 40 | 105 | 0 |
| Acetic anhydride (50 mM) | 4 | 90 | 50 | 40 | 46 | 121 | 30 |
| | 2 | 100 | 70 | 30 | 51 | 134 | 30 |
| | 1 | 100 | 70 | 0 | 54 | 142 | 0 |
| | 0 | 80 | 20 | 0 | 43 | 113 | 0 |
| Glutardialdehyde | 4 | 90 | 60 | 30 | 46 | 121 | 20 |
| + (EDC + methylamine) | 2 | 90 | 60 | 60 | 52 | 137 | 30 |
| (20 mM + 3M) | 1 | 90 | 70 | 10 | 52 | 137 | 0 |
| | 0 | 70 | 60 | 0 | 43 | 113 | 0 |
| Membranes from dimethylsulphate-modified cells | 4 | 100 | 70 | 10 | 55 | 145 | 0 |
| | 2 | 100 | 30 | 0 | 42 | 110 | 0 |

* Membrane equivalents corrected for losses; † Tumour-bearing animals; ‡ No palpable tumours at 90 days.

fraction isolated from viable tumour cells. Control immunizations were performed with non-modified as well as with neuraminidase-treated membrane fractions. To correlate with the most efficient immunizing dosages of modified cells previously reported (Staab and Anderer, 1977) we estimated the losses of membrane material during membrane preparation, and used for immunization only doses of the modified-membrane fractions which were corrected for these losses. In a previous experiment (Staab and Anderer, 1977) the optimal immunizing dose was found to be between $10^2$ and $10^4$ modified cells. Therefore, immunization was now carried out with membrane equivalents corresponding to $10^4$, $10^2$, $10^1$ and $10^0$ cells. The latter values were predominantly considered as controls to characterize the trend of infinitesimal dilution. The amount of membrane protein present in a membrane equivalent of $10^1$ cells was about 1 ng.

The tumour cells used for the challenge were derived from one and the same passage, and exhibited an $LD_{100}$ of $5 \times 10^2$ cells per animal. The challenge dosage of $10^5$ viable tumour cells was given 14 days after immunization. This interval was found to be sufficient for inducing tumour protection when immunization was carried out with modified cells (Staab and Anderer, 1977). In the course of the experiment we determined the number of animals which remained tumour free for 90 days after challenge with viable cells. The mean survival time was calculated only for those mice which developed tumours. The resulting value, measured in days or in percentage of the survival time of the controls, represented an additional criterion for induced immunity against the tumour transplant. The results, together with the number of living animals at Days 30, 50 and 70 after the tumour challenge are given in Table I. The data of some experiments in which the modifying conditions did not improve the immunogenic capacity of the isolated membrane fraction are not listed.

Immunization of mice with unmodified membrane fractions showed at most up to 20% of tumour-free animals, and only a moderate increase in mean survival time. This result is comparable to the findings after immunization of mice with X-irradiated tumour cells (Staab and Anderer, 1977). Treatment of the membrane fraction with neuraminidase did not appear to increase the immunogenic capacity of the sample. The best immunizing effect was found with the membrane fraction modified with dimethylsulphate in a concentration of 50 mM. A 5-fold increase in concentration of the modifying reagent did not improve the immunogenic capacity of the membrane sample. Up to 60% of the animals remained tumour free and the mean survival time of the tumour-bearing animals showed the highest increase over the other experimental groups of mice. Immunization with membrane samples modified with acetic anhydride as well as with glutardialdehyde/(EDC + methylamine) induced a smaller but still significant increase in immunogenicity. With doses of the membrane samples corresponding to $10^2$ and $10^4$ tumour cells, up to 30% of the animals remained tumour free thus exhibiting a slightly better immunizing effect than unmodified membrane samples. A 5-fold increase in acetic anhydride concentration during the modifying reaction did not increase the immunogenicity of the resulting membrane sample. However, considering the concentrations of the reagents of the complex modification with glutardialdehyde/(EDC + methylamine), one has to note that a reduction of the methylamine concentration by a factor 5 led to a less immunogenic membrane sample. In all experiments, no significant differences in the distribution of tumours between male and female animals were observed.

The most effective doses of all the modified-membrane samples were membrane equivalents corresponding to $10^2$–$10^4$ tumour cells. There is a slight tendency that membrane equivalents of $10^2$ cells were more effective in inducing immunity against the tumour-cell transplant. Further reduction of the immunizing dose, however, abolished the immunizing effect.

The immunological specificity of tumour protection could be demonstrated in a number of control experiments. As a non-cross-reacting tumour we used an

TABLE II.—*Protection of STU mice against challenge with* $10^5$ *STU-D 17 cells after pretreatment with methylated or unmodified membrane fractions from STU-D 17 cells (transformed by RSV), from STU-51A/232B cells (transformed by SV 40) and from STU embryonic tissue. The challenge was given 14 days after immunization with a single dose equivalent to* $10^4$ *cells.*

| Immunizing membrane fraction | % Animals alive, 30 days after challenge | 50 | 70 | Mean survival* (% of control) | Tumour-free animals† (% per group) |
|---|---|---|---|---|---|
| Saline buffer | 100 | 50 | 0 | 100 | 0 |
| STU-D 17 | 100 | 50 | 20 | 112 | 0 |
| STU-D 17 methylated | 100 | 100 | 60 | 131 | 40 |
| STU 51A/232B | 100 | 60 | 0 | 103 | 0 |
| STU 51A/232B methylated | 100 | 80 | 0 | 110 | 0 |
| STU foetal tissue | 100 | 60 | 0 | 98 | 0 |
| STU foetal tissue methylated | 100 | 70 | 0 | 109 | 0 |

* Tumour-bearing animals; † no palpable tumours at 90 days.

SV 40-transformed cell line, STU 51A/232B, also derived from embryonic cells of STU mice. Since this cell line was grown in the same culture medium as the STU-D 17 cells taken for challenge, the STU 51A/232B membrane fraction could simultaneously serve as a control for possible immunogenic effects induced by foetal bovine serum components adhering to the cells surface membranes. A crude membrane fraction prepared from foetal tissue of STU mouse embryos should give information on the immunogenic effects of foetal antigens. All these specificity controls were performed only with male mice to exclude non-specific effects based on the presence of sex-linked antigens. The membrane fractions were tested as unmodified samples, as well as samples modified with dimethyl sulphate.

The data listed in Table II again show the highly significant effect of methylation on the specific immunogenicity of the STU-D 17 membrane fraction. Neither immunization with the membrane fraction of the non-cross-reacting tumour cell line STU 51A/232B, nor with that derived from foetal tissue, whether applied in the unmodified or in the methylated form, exhibited any influence on the growth of the STU-D 17 tumour transplants or on the survival of experimental animals. Thus, one can conclude that the immunogenic effect of the methylated STU-D17 membrane fraction is tumour specific.

In order to characterize the type of immune response induced by the modified membrane fractions, a membrane-permeability assay (Kurth and Medley, 1975) was used to detect cytotoxic antibodies. The preimmune sera represented the negative controls, and a rabbit serum obtained after immunization with STU-D 17 tumour cells was used as positive control. In a final serum dilution 1:10 the positive control serum induced 70% membrane permeability, in a dilution 1:20 permeability was 37%. All pooled mouse sera obtained 7 days after the tumour challenge were assayed in a final serum dilution 1:20. In all cases membrane permeability was found to be less than 10% which indicated that the amount of cytotoxic antibodies, if they are present at all, must be very low.

### DISCUSSION

The effects of the individual chemical modifiers on the immunogenicity of the membrane fraction of STU-D 17 tumour cells correlate fairly well with the effects previously observed after immunization with modified tumour cells (Staab and Anderer, 1977). This finding opens up the possibility of inducing comparable tumour protection by immunization without the risk of transmitting biologically hazardous genetic information. Optimal tumour protection and increased survival time were again found to depend on the chemical nature of the modifying group as well as on the dose of the immunizing membrane fraction. Modification of the membrane fraction with dimethylsulphate induced the most efficient alteration of immunogenicity as against the non-modified membrane sample. On the other hand, best results were obtained with the lower immunizing doses, *i.e.* with membrane equivalents of $10^2$ cells. The controls of infinitesimal dilution of the immunizing material clearly showed that tumour protection was specifically bound to the immunizing membrane sample. The experimental manipulation of animals during immunization scarcely has any effect, since the survival time of all groups of mice receiving a membrane dose equivalent to $10^0$ cells was comparable to that of the non-immunized control group.

The membrane antigens responsible for tumour rejection were found to be distinctly STU-D 17-tumour-specific. Immunogenic effects of contaminating antigens derived from foetal bovine serum of the culture medium, which might adhere to the cell surface and remain adhered during membrane preparation (Phillips and Perdue, 1977) could be excluded. In control experiments the membrane fraction of a non-cross-reacting tumour cell line,

STU 51A/232B, grown in the same culture medium did not induce any tumour rejection. Neither did mouse foetal antigens present on the surface of STU-D 17 cells appear to participate in tumour rejection, since immunization with membrane fractions prepared from foetal tissue did not influence the growth of STU-D 17 tumour transplants. Sex-linked surface antigens responsible for tumour rejection can also be ruled out, since the distribution of male and female animals within the groups of survivors was about equal.

The failure to produce an adequate immune response observed with the membrane fraction isolated from dimethylsulphate-modified tumour cells, is difficult to explain. A possible explanation is offered by the assumption that methylated cell membranes preferably form "inside out" vesicles during membrane isolation. This would lead to a drastic decrease in accessible sites which are relevant for a specific immune response against the cell-surface pattern. On the other hand, it cannot be excluded that rearrangement of the methylated membrane during isolation may occur, possibly including conformational shifts in the methylated membrane proteins, thus altering the immunogenic specificity of the surface structures which are originally responsible for the effect of tumour protection.

In the light of these results, one may expect that chemical modification of isolated tumour-specific surface antigens might still improve the immune response specific for tumour protection.

The authors thank Dr H. P. Kulas for performing the iodination experiment and Miss S. Glock for excellent technical assistance.

### REFERENCES

CHAMPLIN, R. & HUNTER, R. L. (1975) Studies on the composition of adjuvants which selectively enhance delayed-type hypersensitivity to lipid conjugated antigens. *J. Immunol.*, **114**, 76.

CHAO, H. F., PEIPER, S. C., AACH, R. D. & PARKER, C. W. (1973) Introduction of cellular immunity to a chemically altered tumour antigen. *J. Immunol.*, **111**, 1800.

Committee on standardized nomenclature for inbred strains of mice (1968) Standardized nomenclature for inbred strains of mice: fourth listing. *Cancer Res.*, **28**, 391.

COON, J. & HUNTER, R. (1972) Selective induction of delayed hypersensitivity by a lipid conjugated protein antigen which is localized in thymus-dependent lymphoid tissue. *J. Immunol.*, **110**, 183.

DULBECCO, R. & FREEMAN, C. (1959) Plaque production by the polyoma virus. *Virology*, **8**, 396.

HUBBARD, A. L. & COHN, Z. A. (1975) Externally disposed plasma membrane proteins: I. enzymatic iodination of mouse L cells. *J. Cell Biol.*, **64**, 438.

HUNTER, R. L. & STRICKLAND, F. (1975) Immunization with a lipid-conjugated membrane antigen to suppress growth of fibrosarcoma induced by simian virus 40. *J. natn. Cancer Inst.*, **54**, 1157.

KULAS, H. P., MARGGRAF, W. D., KOCH, M. A. & DIRINGER, H. (1972) Comparative studies of lipid content and lipid metabolism of normal and transformed mouse cells. *Hoppe-Seyler's Z. Physiol. Chem.*, **353**, 1755.

KURTH, R. & MEDLEY, G. (1975) A membrane permeability test for the detection of cell surface antigens. *Immunology*, **29**, 803.

LOWRY, O. H., ROSEBROUGH, N. J., FARR, A. L. & RANDALL, R. J. (1951) Protein measurement with the folin phenol reagent. *J. biol. Chem.*, **193**, 265.

PARISH, C. R. (1971*a*) Immune response to chemically modified flagellin: I. Induction of antibody tolerance to flagellin by acetoacetylated derivatives of the protein. *J. Exp. Med.*, **134**, 1.

PARISH, C. R. (1971*b*) Immune response to chemically modified flagellin: II. Evidence for a fundamental relationship between humoral and cell-mediated immunity. *J. Exp. Med.*, **134**, 21.

PARISH, C. R. (1973) Immune response to chemically modified flagellin: IV. Further studies on the relationship between humoral and cell-mediated immunity. *Cell Immunol.*, **6**, 66.

PARISH, C. R. & LIEW, F. Y. (1972) Immune response to chemically modified flagellin: III. Enhanced cell-mediated immunity during high and low zone antibody tolerance to flagellin. *J. Exp. Med.*, **135**, 298.

PHILLIPS, E. R. & PERDUE, J. F. (1977) Immunologic identification of fetal calf serum-derived proteins on the surfaces of cultured transformed and untransformed rat cells. *Int. J. Cancer*, **20**, 789.

PRAGER, M. D. & BRAECHTEL, F. S. (1973) Methods for modification of cancer cells to enhance their antigenicity. In *Methods in Cancer Research*. Vol. 9 Ed. H. Busch. New York: Academic Press. p. 339.

SCHIRRMACHER, V. & WIGZELL, H. (1972) Immune response against native and chemically modified albumins in mice. *J. Exp. Med.* **136**, 1616.

STAAB, H. J. & ANDERER, F. A. (1976) Structure and immunogenic behaviour of methylated tobacco mosaic virus. *Biochim. Biophys. Acta*, **427**, 453.

STAAB, H. J. & ANDERER, F. A. (1977) Immunogenicity of tumour cells modified with various chemicals. *Br. J. Cancer*, **35**, 395.

THOMSON, K., HARRIS, M. & BENJAMINI, E. (1972) Cellular and humoral immuity: A distinction in antigenic recognition. *Nature New. Biol.*, **238**, 20.

VASUDEVAN, D. M., BALAKRISHNAN, K. & TALWAR, G. P. (1970) Effect of neuramiidase on electrophoretic mobility and immune cytolysis of human uterine cervix carcinoma cells. *Int. J. Cancer* **6**, 506.